# Newcastle Disease Virus Vectored Chicken Infectious Anaemia Vaccine Induces Robust Immune Response in Chickens

**DOI:** 10.3390/v13101985

**Published:** 2021-10-02

**Authors:** Madhan Mohan Chellappa, Sohini Dey, Dinesh Chandra Pathak, Asmita Singh, Narayan Ramamurthy, Saravanan Ramakrishnan, Asok Kumar Mariappan, Kuldeep Dhama, Vikram N. Vakharia

**Affiliations:** 1 Recombinant DNA Laboratory, Division of Veterinary Biotechnnology, Indian Veterinary Research Institute, Bareilly 243122, UP, India; dennisdinesh16@gmail.com (D.C.P.); asmitaivri@gmail.com (A.S.); vetnarayan007@gmail.com (N.R.); 2 Immunology Section, Indian Veterinary Research Institute, Bareilly 243122, UP, India; dearsaromib@yahoo.com; 3 Division of Pathology, Indian Veterinary Research Institute, Bareilly 243122, UP, India; drasokvet@gmail.com (A.K.M.); kdhama@rediffmail.com (K.D.); 4 Institute of Marine and Environmental Technology, University of Maryland Baltimore County, Baltimore, MD 21202, USA; vakharia@umbc.edu

**Keywords:** Newcastle disease, chicken infectious anaemia, bivalent vaccine, reverse genetics, immune response, challenge study

## Abstract

Newcastle disease virus (NDV) strain R2B, with an altered fusion protein cleavage site, was used as a viral vector to deliver the immunogenic genes VP2 and VP1 of chicken infectious anaemia virus (CIAV) to generate a bivalent vaccine candidate against these diseases in chickens. The immunogenic genes of CIAV were expressed as a single transcriptional unit from the NDV backbone and the two CIA viral proteins were obtained as separate entities using a self-cleaving foot-and-mouth disease virus 2A protease sequence between them. The recombinant virus (rR2B-FPCS-CAV) had similar growth kinetics as that of the parent recombinant virus (rR2B-FPCS) in vitro with similar pathogenicity characteristics. The bivalent vaccine candidate when given in specific pathogen-free chickens as primary and booster doses was able to elicit robust humoral and cell-mediated immune (CMI) responses obtained in a vaccination study that was conducted over a period of 15 weeks. In an NDV and CIAV ELISA trial, there was a significant difference in the titres of antibody between vaccinated and control groups which showed slight reduction in antibody titre by 56 days of age. Hence, a second booster was administered and the antibody titres were maintained until 84 days of age. Similar trends were noticed in CMI response carried out by lymphocyte transformation test, CD4^+^ and CD8^+^ response by flow cytometry analysis and response of real time PCR analysis of cytokine genes. Birds were challenged with virulent NDV and CIAV at 84 days and there was significant reduction in the NDV shed on the 2nd and 4th days post challenge in vaccinated birds as compared to unvaccinated controls. Haematological parameters comprising PCV, TLC, PLC and PHC were estimated in birds that were challenged with CIAV that indicated a significant reduction in the blood parameters of controls. Our findings support the development and assessment of a bivalent vaccine candidate against NDV and CIAV in chickens.

## 1. Introduction

Chicken infectious anaemia infection and Newcastle disease are two economically important diseases of chickens that are prevalent worldwide and cause heavy economic losses to the poultry industry. Chicken infectious anaemia infection is caused by chicken infectious anaemia virus (CIAV), which is a non-enveloped virus with icosahedral symmetry, belonging to the Circoviridae family. CIAV is one of the smallest viruses, with a diameter of 25 nm that encloses a single negative-stranded circular DNA of 2.3 kb size. CIAV genome encodes for three proteins; namely, VP1, VP2 and VP3 [1,2,3]. VP1 protein is the largest among all encoded proteins, having a size of 54 kDa. It is the only known protein to be involved in capsid formation of CIAV. The N-terminal region of VP1 is rich in positive charged amino acids such as arginine, which assists VP1 protein in binding with DNA. VP2 protein shows phosphatase activity on serine, threonine and tyrosine phosphatases of both VP1 and VP3, and because of this activity, it acts as a scaffold protein for VP1 to attain its proper conformation. Synchronous synthesis of both VP1 and VP2 proteins in the same cell is a must for the generation of a conformational neutralising epitope [4].

Newcastle disease is caused by Newcastle disease virus (NDV), which is an enveloped virus belonging to the Paramyxoviridae family. The virus is encoded by six proteins; namely, NP, P, M, F, HN and L. Chicken infectious anaemia virus causes clinical signs such as anorexia, weakness, stunting growth and thriftiness, weight loss and anaemia that can be observed externally, whereas intramuscular haemorrhages, lymphoid atrophy and bone marrow aplasia are mostly seen in histopathology among young chicks. Newcastle disease virus causes symptoms that can be classified as either enteric or neurological in nature. Newcastle disease virus strain R2B is a mesogenic virus that is used as a vaccine strain in many of the endemic Asian countries. The complete genome size of R2B is 15,186 nucleotides and the reverse genetic system for this virus has been established in our laboratory [5,6,7]. The virus strain R2B, which is moderately virulent in nature, cannot be used for vaccination purposes in young chicks less than six weeks old. Hence the amino acids at the fusion protein cleavage site of the virus, which are a major determinant of the virulence, have been modified to resemble a lentogenic backbone and they have been rescued recently [8]. The rescued viruses in our laboratory have been used in designing vaccine candidates against avian reovirus and rabies virus and have been shown to work in multiple host species [9,10].

The current CIAV vaccines include live attenuated [11], inactivated [12], DNA vaccines [13,14,15] and subunit vaccines generated from bacterium, *Escherichia coli* [16,17] or eukaryotic baculoviral system [4,18,19,20]. Live vectored vaccines based on reverse genetics are currently in vogue against many diseases in several countries. NDV reverse genetics system has been proven to be used as a live vector to deliver immunogenic genes of many important poultry viral diseases. The present study involves using the NDV backbone in developing a bivalent vaccine candidate against chicken infectious anaemia and Newcastle disease in chickens.

## 2. Materials and Methods

### 2.1. Viruses, Cells and Specific Pathogen-Free (SPF) Eggs

Virulent Newcastle disease virus isolate was characterised in our laboratory and its nucleotide sequence is available under the accession number MF422127.1. An Indian isolate of virulent chicken infectious anaemia virus was obtained from the Avian Diseases section, Indian Veterinary Research Institute, grown in MSB-1 cell culture, and its nucleotide sequence is available under the accession number AY583758.1. Characterised Newcastle disease virus strain R2B, with modified fusion protein cleavage site (FPCS), was rescued in our laboratory [8]. Vero cells were obtained from National Centre for Cell Sciences, Pune, India and were maintained in Medium 199 supplemented with 10% heat-inactivated foetal bovine serum maintained at 37 °C with 5% CO_2_. SPF eggs were obtained from Venkateshwara Hatcheries Pvt. Ltd., Pune, India and were incubated in the egg hatcher cum incubator until the SPF chicks were obtained.

### 2.2. Generation of Recombinant NDV Containing the VP2 and VP1 Genes of CIAV

The full-length infectious clone of NDV-R2B with the altered cleavage site along with the three support plasmids pNP, pP and pL are available in the laboratory. The transcriptional cassette containing the immunogenic genes of CIAV was introduced within the P and M genes of the full-length infectious clone. The immunogenic genes of CIAV, comprising complete ORF of VP2 and VP1, were chemically synthesised as a single gene construct having a foot-and-mouth disease virus (FMDV) 2A protease gene between them (GenScript, Piscataway, NJ, USA). The order of the immunogenic genes in the synthetic construct comprised VP2–FMDV-2A–VP1. The stop codon of VP2 was removed and the FMDV-2A was fused with the VP2, followed by the start codon of VP1. The sequence of FMDV-2A is CAGCTGTTGAATTTTGACCTTCTTAAGCTTGCGGGAGACGTCGAGTCCAACCTGGGC. The entire immunogenic gene construct was preceded by a Kozak sequence GCCACC. One extra nucleotide ‘T’ was added at the end of the stop codon of VP1 to maintain the ‘rule of six’. The ORF of VP2 without a stop codon is 648 nucleotides and that of VP1 is 1350 nucleotides. The entire transcriptional cassette was introduced into the backbone of NDV by directional cloning using the restriction sites *PacI* and *AvrII*. 

Rescue of recombinant NDV along with the immunogenic genes of CIAV was achieved in Vero cells.The full-length clone of the virus and the support plasmids were used for transfection. The Vero cells were grown in six-well plates and transfection was carried out after the cells attained 80% confluency. The media was removed and the cells were flooded with optiMEM media and kept at 37 °C for one hour. The plasmids pNDV–VP2 + VP1 (1.5 µg), pNP (0.6 µg), pP (0.3 µg) and pL (0.2 µg) were diluted in optiMEM medium in which Lipofectamine 3000 reagent was added and incubated for 30 min according to manufacturer’s instructions (Invitrogen, Carlsbad, CA, USA). The optiMEM media were removed from the virus cells and the plasmid–Lipofectamine mixture was added on to the cells in a drop wise manner. Further 1.0 mL of optiMEM media was added to the cells along with acetyl trypsin (1 µg/_mL_) and was left undisturbed at 37 °C incubator with 5% CO_2_ for 72 h. After the incubation period, the transfected mixture was removed and transferred to another set of fresh Vero cells. Ten serial passages were given and the recombinant virus was analysed by RT-PCR. 

The stability of the recombinant virus was confirmed by serially passaging the virus in SPF eggs 10 times and analysing it for its stability. RNA from the recombinant virus present in the allantoic fluid was isolated by TriZOL reagent and an RT-PCR was carried out using gene-specific primers for NDV as well as CIAV.

### 2.3. Characterisation of Recombinant Viruses

Characterisation of recombinant viruses was carried out by a combination of molecular and biological methodologies. The recombinant NDV virus containing the CIAV genes was purified by ultra-centrifugation using established protocols [21]. The purified virus was characterised in a Western blot by using anti-NDV polyclonal serum (Abcam, Cambridge, MA, USA) and anti-CIAV polyclonal serum (USB Biologicals, Salem, MA, USA).

### 2.4. Fluorescence Assay

A multiplicity of infection of 0.01 (MoI) of the recombinant virus was used to infect 80 % confluent Vero cells. Both the normal and infected cells after 48 hrs of incubation at 37 °C were washed with PBS and fixed with 4% paraformaldehyde for 90 min at 37 °C. Following the incubation period, cells were treated with 0.5% Triton-X100 for 10 min at room temperature to permeabilise the membrane. The cells were then blocked with 5% bovine serum albumin for 30 min at room temperature. They were washed three times with PBS and incubated for 1 h with a mixture of anti-NDV and anti-CIAV primary antibodies, as mentioned previously [9]. At the end of incubation period, cells were washed thrice with PBS and incubated with a mixture of FITC labelled rabbit anti-chicken IgG and Alexa Fluor 568 labelled goat anti-chicken IgG and kept for one hour at room temperature. Nucleus staining was performed using DAPI at 1:1000 dilution and finally the cells were visualised for corresponding fluorescence a images were taken using Fluoview FV 1000 confocal microscope (Olympus, Tokyo, Japan) at the matching excitation and emission filters for FITC and Alexa Fluor 568, as described [9].

Growth patterns of the recombinant viruses were evaluated by multi cycle of growth conditions in monolayers of Vero cells in six-well plates at an MOI of 0.01. The growth kinetics study was performed as per established protocols and the titles of the virus were calculated using the standard method [9]. Further, titration of the recombinant viruses was expressed as 50% tissue culture infectious dose and was carried out by established protocols [22].

The biological characterisation of the recombinant virus was evaluated by mean death time (MDT) in 9–11 days old SPF eggs and intra-cerebral pathogenicity index (ICPI) analysis in day-old SPF chickens, as per standard procedures [23].

### 2.5. In Vivo Animal Experimentation Studies

The animal experiments involving SPF chickens were conducted in accordance with the rules and regulations as laid down by the Institute Animal Ethics Committee, Indian Veterinary Research Institute (F.No.26-1/2020-21/JD(R)/IAEC dated 30 September 2020).

**Figure 1:**
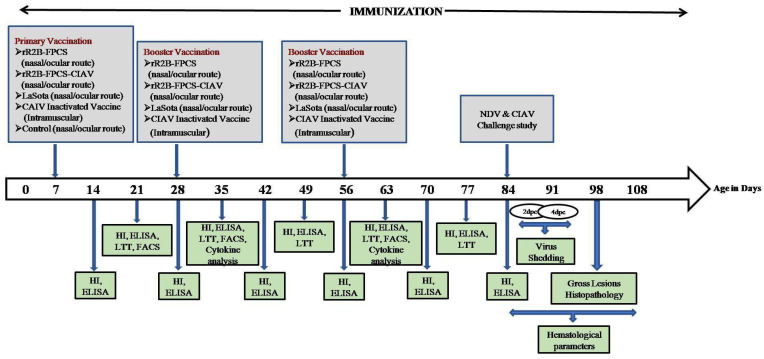
Schematic diagram showing the experimental design. CIAV: Chicken infectious anaemia virus, NDV: Newcastle disease virus, ELISA: enzyme-linked immunosorbent assay, HI: haemagglutination inhibition test, LTT: lymphocytes transformation test, FACS: fluorescent-activated cell sorter, dpc: days post challenge.

The immunisation and protection studies of the vaccine candidate were evaluated in SPF chicks which were maintained under standard managerial practices and were provided with autoclaved feed and water throughout the study period. The schematic diagram showing the experimental design is presented in Figure 1.

A total of 66 SPF chicks were divided into five different groups (*n* = 10) with 26 birds in the control group (10 birds each for challenge with virulent NDV and CIAV and 6 birds remained unchallenged to be used in histopathological study). The birds were inoculated with 1 × 10^6^ TCID_50_/_mL_ each of the rR2B-FPCS and rR2B-FPCS-CIAV vaccines, 1 × 10^6^ EID_50_/_mL_ of LaSota vaccine and 10^4^ TCID_50_/_mL_ of CIAV-inactivated oil-adjuvanted vaccine, according to manufacturer’s recommendations. The sera samples from the pre-immunised and post-immunised chicken were collected from all the groups at weekly intervals. Humoral immune response was evaluated by haemagglutination inhibition test and enzyme-linked immunosorbent assay for checking the antibody status against NDV using recombinant nucleoprotein as described earlier [24] and CIAV using chicken anaemia virus antibody test kit (IDEXX, Westbrook, ME, USA). CMI response was evaluated in peripheral blood mononuclear cells (PBMCs) by antigen-specific lymphocyte transformation test and gene expression analysis of cytokines; namely, IL-1β, IL-12, IFN-γ, IL-10 and IL-4 by real-time PCR analysis, as described earlier [24]. Similarly, the immune cells of the vaccinated birds were also analysed by flow cytometry (BD Biosciences, San Jose, CA, USA) to determine the expression level of CD4^+^ and CD8^+^ T lymphocyte cells, as per earlier studies [8]. The PBMCs (1 × 10^6^ cells) were incubated with 2 µg anti-chicken CD3-FITC labelled, 0.4 µg each of anti-chicken CD4^+^-RPE labelled and anti-chicken CD8^+^-Cy5 labelled conjugates (Southern Biotech, Birmingham, AL, USA) for estimation.

Furthermore, the birds were challenged on the 84th day with virulent NDV and CIAV (10^5^ EID_50_/_mL_ and 10^4.5^ TCID_50_/_mL_, respectively) intramuscularly. Birds were daily observed up to 14 days post challenge (dpc) for clinical signs of NDV and surviving chickens were humanely sacrificed. Virus shedding study for NDV was carried out on inoculation into 9-day-old SPF embryonated chicken eggs and a haemagglutination assay was conducted as described earlier [8]. Similarly, the birds were observed up to 25 dpc and various haematological parameters such as PCV, TLC, PLC and PHC were estimated in birds that were challenged with CIAV.

### 2.6. Haematology

Haematological parameters were evaluated at 5, 10, 15, 20 and 25 dpc using blood collected from at least 3 birds per group. The blood was collected in heparinised vacutainers for analysing various parameters. Packed cell volume (PCV) (micro-haematocrit capillary tube method), haemoglobin (Sahli’s Method), total erythrocyte count (TEC), total leucocyte count (TLC) and differential leucocyte count (DLC) were performed as per the standard procedure [25]. Peripheral lymphocyte count (PLC) and peripheral heterophil count (PHC) were derived from the DLC as per the previously reported method [26].

### 2.7. Histopathology and Lesion Scoring

The representative tissue samples of conjunctival mucous membrane, thymus, oesophagal-proventricular junction, Peyer’s patches, spleen, caecal tonsils, bursa of Fabricius and liver were collected in 10% neutral buffered formalin. Paraffinised tissue blocks were made from formalin-fixed tissues that were processed through graded ethanol, xylene, paraffin embedding and paraffin tissue blocking. The tissue blocks were subjected to microtomy to obtain 5 μm thick sections. The obtained sections were stained with haematoxylin and eosin (H&E) stain for histopathological examination following the standard protocol [27]. Lesion scoring of the immune organs was carried out as per procedure [28].

### 2.8. Statistical Analysis

Statistically significant differences in data from different vaccinated groups at different time points were evaluated by two-way analysis of variance (ANOVA). Tukey’s multiple comparison test was used and values below 0.0001 were considered significant for all the analyses. Statistical analysis was conducted using Prism 7.0 computer software (Graphpad Software Inc., San Diego, CA, USA).

## 3. Results

### 3.1. Generation of Recombinant NDV Containing the Immunogenic Genes of CIAV

**Figure 2:**
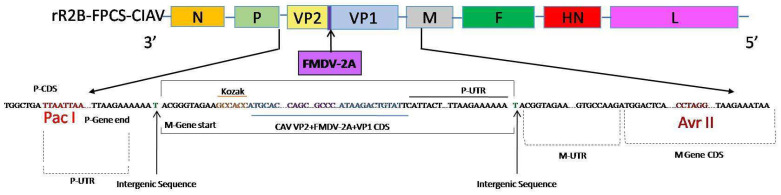
The NDV-R2B-FPCS backbone generated by reverse genetics contained the altered FPCS amino acid motif similar to that of a lentogenic strain of NDV. The VP2/VP1 gene cassette of CIAV was introduced between the P and M genes bound by restriction enzymes *Pac*I and *Avr*II. A 60 nucleotides FMDV 2A protease sequence was introduced between the VP2 and VP1 of CIAV. P CDS: P gene coding sequence; P-UTR: P gene non-coding region; M-UTR: M gene non-coding region; M Gene CDS: M gene coding sequence.

The transcription cassette containing the VP2 + FMDV-2A + VP1 genes was flanked by the NDV transcriptional signals in the form of gene start and gene end regulatory elements and was inserted in the intergenic region between the P and M genes of NDV backbone. The generated full-length clone thus obtained was designated as rR2B-FPCS-CIAV (Figure 2). The total size of the full-length clone was 17,466 nucleotides which maintain the rule of six. Rescue of the virus in Vero cells was evidenced by the appearance of syncytia in the Vero cells when supplemented with acetyl trypsin. No syncytia were observed in control cells. Sequence analysis of the RT-PCR products obtained from the viral RNA confirmed the intactness and precise junctions of the NDV and CIAV genes in the transcription cassette and its expression was stable even after 10 serial egg passages.

### 3.2. Molecular and Biological Characterisation of rR2B-FPCS-CIAV Virus

**Figure 3:**
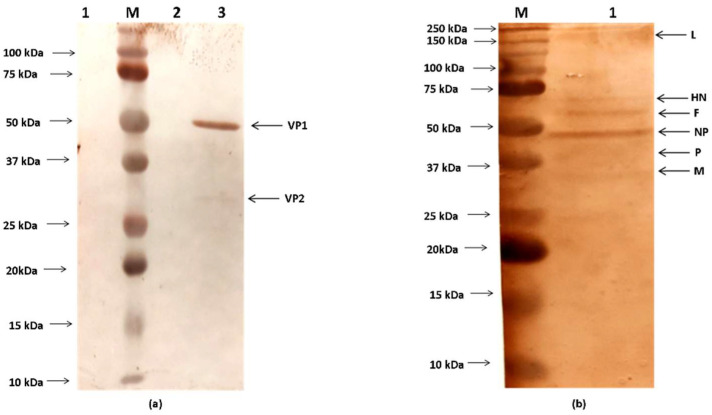
Western blot analysis of the purified recombinant NDV containing VP2/VP1 gene of CIAV. (**a**) Lane 1: Vero cell control; Lane 2: vector (rR2B-FPCS) control; Lane 3: the position of the VP2 and VP1 proteins of CIAV; approximately 27 kDa and 52 kDa, respectively, in the recombinant virus (rR2B-FPCS-CIAV) and (**b**) Lane 1: the positions of the NDV proteins; namely, L, HN, F, NP, P and M along with the protein marker in Lane M (Precision Plus Protein Standards, Bio-Rad, USA) are indicated.

**Figure 4:**
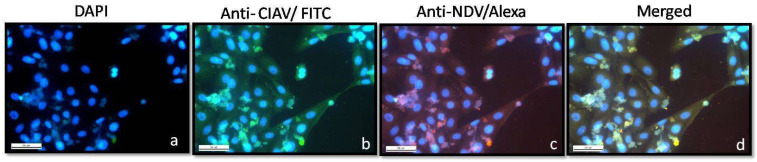
Immunofluorescence analysis of rNDV-R2B-CIAV expressing VP2/VP1 protein of chicken infectious anaemia virus ×60. (**a**) DAPI-stained cells; (**b**) cells expressing VP2/VP1 of CIAV (anti-CIAV/FITC); (**c**) cells expressing NDV proteins (anti-NDV/Alexa); (**d**) merged. (Scale bar: 50 µm).

Western blot analysis of the purified virus after the fractionation by SDS-PAGE revealed the presence of all the six proteins of NDV and the two immunogenic proteins of CIAV on reactivity with specific antisera against NDV and CIAV, respectively (Figure 3). Similarly, the immunofluorescence profile revealed the presence of rR2B-FPCS-CIAV in Vero cells at 48 h post infection (hpi), as evidenced by the presence of red and green fluorescence in the cells (Figure 4).

The MDT score of rR2B-FPCS-CIAV was >168 h and that of the ICPI value was 0.0. Biological characteristics of the rescued rR2B-FPCS-CIAV virus were established via multi cycle growth kinetics in Vero cells. The growth kinetics of the virus revealed the rR2B-FPCS-CIAV virus titres to be 4.2 log10 TCID_50_/_mL_, 6.2 log10 TCID_50_/_mL_ and 7.0 log10 TCID_50_/_mL_ at 12, 24 and 36 hpi and reached the highest titre by 48 hpi and was found to be (7.8 log10 TCID_50_/_mL_). Further, the titres gradually reduced to 7.4 log10 TCID_50_/_mL_ and 7.2 log10 TCID_50_/_mL_ at 60 and 72 hpi. The corresponding titres for the rR2B-FPCS virus at the same time points include 4.5 log10 TCID_50_/_mL_, 6.6 log10 TCID_50_/_mL_, 8.0 log10 TCID_50_/_mL_, 8.2 log10 TCID_50_/_mL_, 7.8 log10 TCID_50_/_mL_ and 7.6 log10 TCID_50_/_mL_, which were lower compared to the rR2B-FPCS virus which was 8.2 log10 TCID_50_/_mL_.3.3. This provided assessment of immunogenicity and protective efficacy of the recombinant viruses against virulent NDV and CIAV challenges in chickens.

The efficacy of the recombinant viruses, rR2B-FPCS-CIAV and rR2B-FPCS, was assessed and compared with commercial vaccines, LaSota for NDV and CIAV-inactivated vaccine for CIAV.

### 3.3. Humoral Immune Response

**Figure 5:**
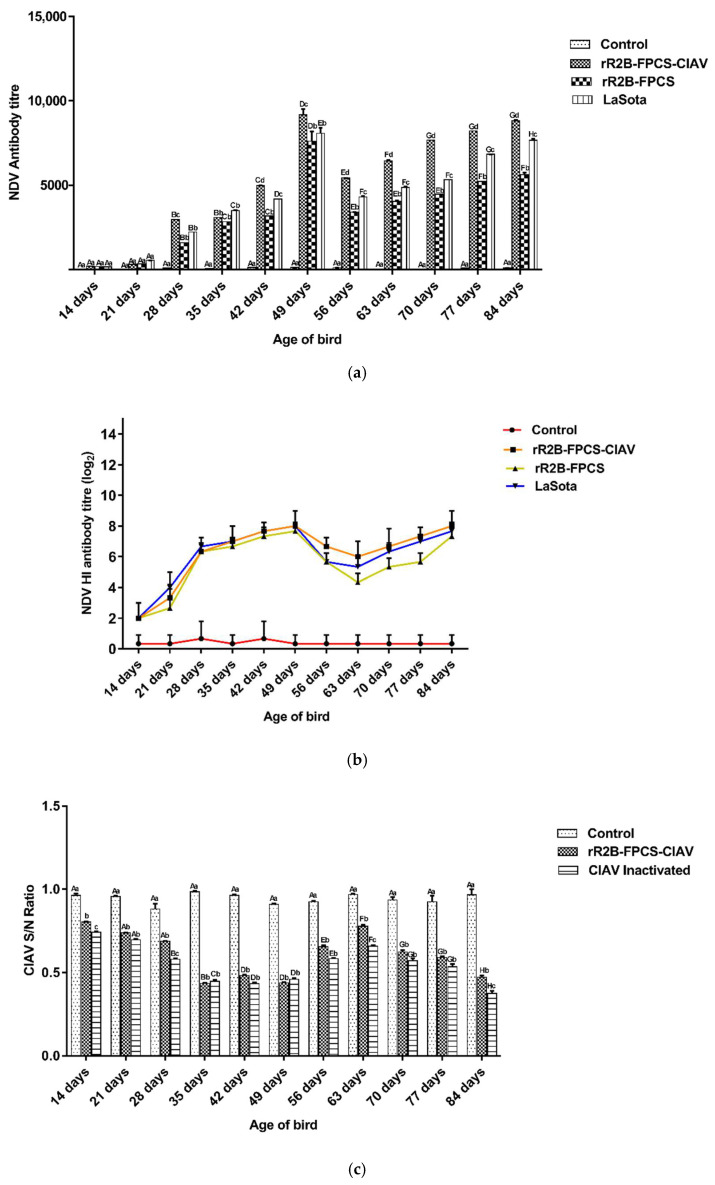
NDV and CIAV specific serum antibody response in the experimental chicken. (**a**) At seven days of age, the birds from three groups were immunised with rR2B-FPCS-CIAV, rR2B-FPCS and LaSota as a primary vaccine. The control group was injected with phosphate buffered saline (PBS). The booster dose was given at 28 and 56 days of age, respectively. At weekly intervals, blood sera were collected from the immunised and control group of birds for testing NDV-specific antibodies by ELISA. The antibody titres higher than 200 were considered positive; (**b**) determination of NDV-specific antibody titre by HI. ELISA and HI were performed at 14, 21, 28, 35, 42, 49, 56, 63, 70, 77 and 84 days of age from all birds. All HI titres were expressed as mean reciprocal of log2 titre ± SEM (standard error of the mean) (*n* = 10); (**c**) one group of birds was vaccinated with CIAV-inactivated vaccine at 7 days of age as a primary vaccine and 28 and 56 days of age as booster dose, respectively. Blood sera collected from the immunised and control group of birds at same intervals were tested for anti-CIAV antibody. The presence of antibody to CIAV was determined by sample to negative (S/N) ratio < 0.6 for each sample. Bars (mean ± SE) indicate the representative data of a single experiment. Data with different capital letter superscripts indicate time effect (*p* < 0.0001) and small letter superscripts indicate the treatment effect (*p* < 0.0001).

Humoral immunity was evaluated in the SPF birds in response to vaccination by ELISA (Figure 5a) and HI assay (Figure 5b) for detection of NDV-specific antibodies at weekly intervals commencing 14 days till 84 days of age of the bird. Similarly, antibodies against CIAV were determined by ELISA (IDEXX, Westbrook, ME, USA) at the same time points (Figure 5c).

A significant difference in the antibody titres was observed between the vaccinated and unvaccinated birds (*p* < 0.0001). A maximal titre was observed at 49 days when a booster was given at 28 days of age. A second booster dose was administered to the birds after they showed a decrease in titre at 56 days. The titre reached a maximum by 84 days and a challenge study was conducted to determine the protective efficacy of the vaccines.

For CIAV, the presence of antibody was determined by the sample to negative (S/N) ratio ≤ 0.6 for each sample. A similar trend was noticed in the progression of antibody titres as compared to NDV ELISA (*p* < 0.0001).

### 3.4. Cell-Mediated Immune (CMI) Response

The CMI response was evaluated by antigen-specific lymphocyte proliferation as determined by lymphocyte transformation test, cytokine gene expression analysis and flow cytometry assay of the activated PBMCs.

**Figure 6:**
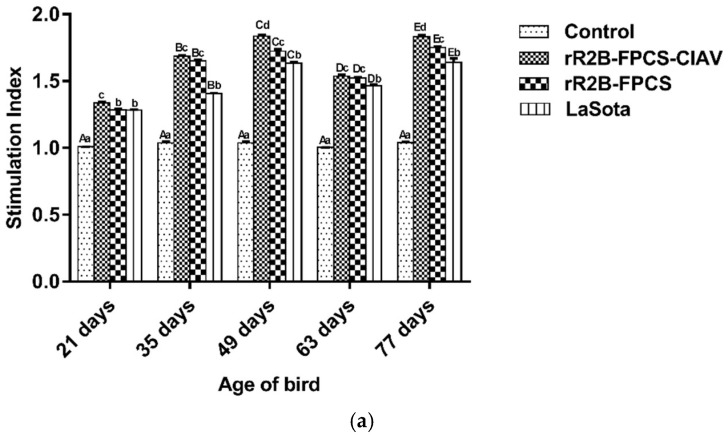

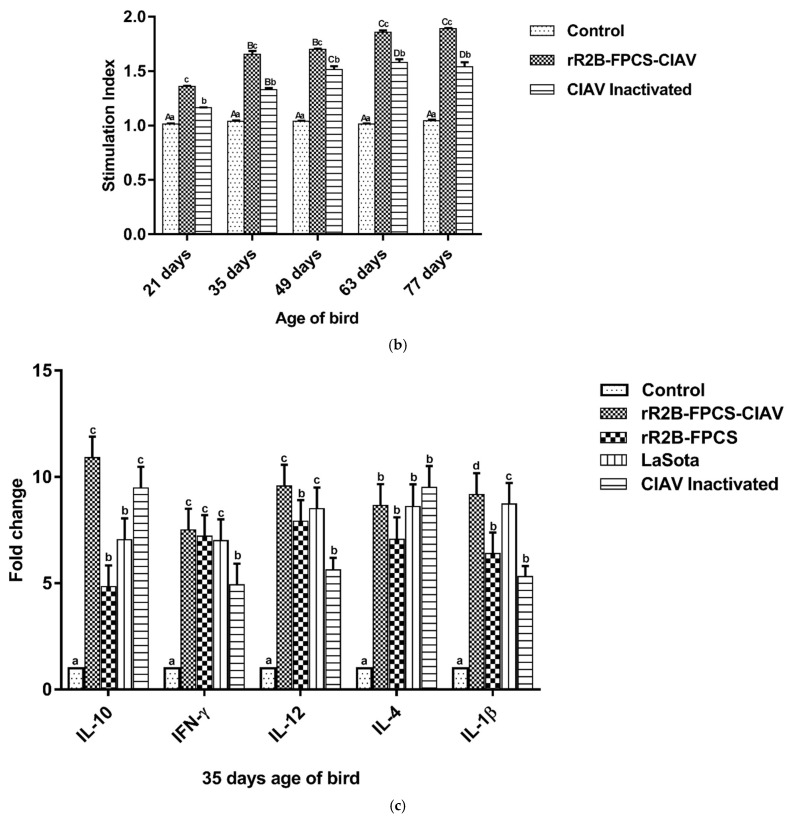

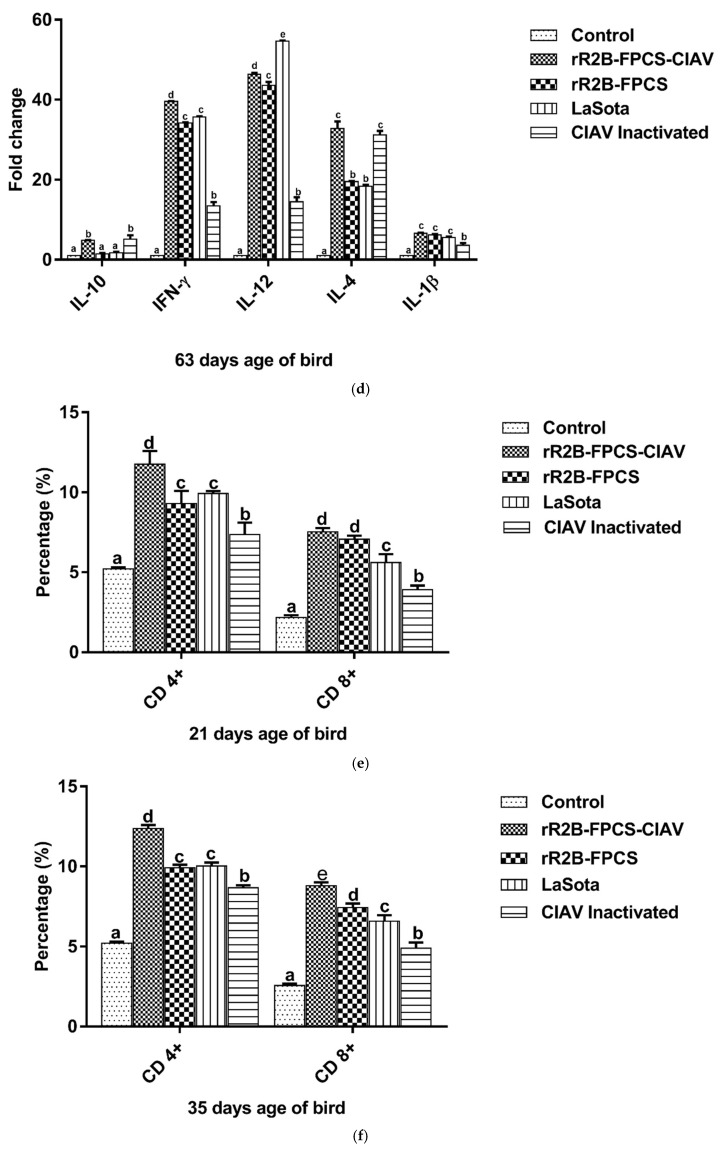

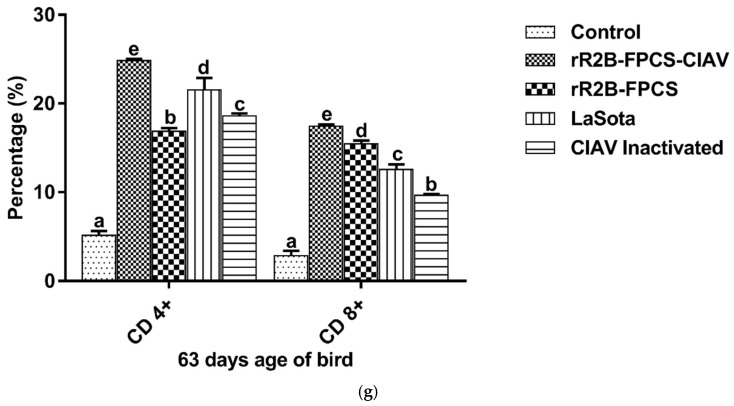
Assessment of (**a**) NDV recombinant NP antigen and (**b**) recombinant CIAV antigen expressed in *Saccharomyces cerevisiae* specific, lymphocyte proliferation response in chicken at 21, 35, 49, 63 and 77 days of age. The PBMCs from the experimental birds (*n* = 10) were stimulated and antigen specific lymphocyte proliferation was expressed as stimulation index (mean ± SE). Different capital letter superscripts indicate the time effect (*p* < 0.0001) while small letter superscripts indicate the treatment effect (*p* < 0.0001); (**c**) at 35 days of age and (**d**) at 63 days of age vaccination induced mRNA level of cytokine genes was measured by quantitative real-time PCR with normalization to GAPDH gene and relative fold change determination by the 2^−ΔΔct^ method. Relative fold change of cytokine gene transcripts is presented. Data are presented as mean value ± standard error and different small letter superscripts indicate the treatment effect (*p* < 0.0001). Flow cytometric analysis for CD4^+^ and CD8^+^ T cell subsets in different vaccinated groups. Percentage of CD4^+^ and CD8^+^ T cells in PBMCs at (**e**) 21 days, (**f**) 35 days and (**g**) 63 days of age of birds in different groups. Bars (mean ± SE) indicate the representative data of a single experiment and data with different small letter superscripts indicate the treatment effect (*p* < 0.0001).

The antigen-specific lymphocyte proliferation was performed at 21, 35, 49, 63 and 77 days of age of the birds. A significantly higher lymphocyte proliferation in the rR2B-FPCS-CIAV-vaccinated birds was observed when compared to other vaccinated groups against NDV (Figure 6a) and CIAV-specific recombinant antigens (Figure 6b) respectively.

The messenger RNA (mRNA) expression of the different cytokine genes, namely, IL-1β, IL-12, IFN-γ, IL-10 and IL-4, was determined at 35 and 63 days of age of birds by quantitative real-time PCR. The levels of cytokines were significantly higher as compared to unvaccinated controls at 35 days of assessment. IL-10 was observed at a significantly higher level in rR2B-FPCS-CIAV and CIAV-inactivated groups followed by LaSota and rR2B-FPCS groups. Moreover, rR2B-FPCS-CIAV was capable of showing Th1-associated responses with an increased expression of IFN-γ, IL-1β and IL-12 genes as compared to other vaccinated groups (*p* < 0.0001) (Figure 6c). A similar trend was noticed at 63 days of assessment in birds except for IL-12 levels which were the highest in the LaSota vaccine group (*p* < 0.0001) (Figure 6d).

On 21, 35 and 63 days of age of the bird, PBMCs from both vaccinated and control groups were analysed for CD4 and CD8 T cell subsets. Birds from vaccinated groups showed significantly higher CD4^+^ (*p* < 0.0001) and CD8^+^ (*p* < 0.0001) T cells than the control group during the days of analysis (Figure 6e–g). Moreover, during the analysis period, rR2B-FPCS-CIAV showed the highest percentage of CD4^+^ and CD8^+^ T cells.

### 3.5. Challenge Studies

**Figure 7:**
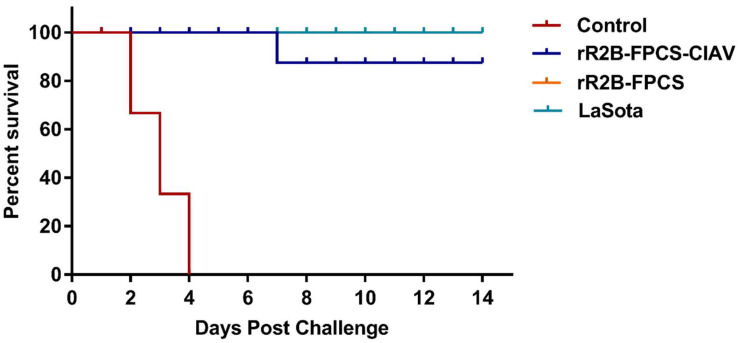
Survival curve for birds immunised with rR2B-FPCS-CIAV, rR2B-FPCS and LaSota. Birds were challenged with 10^5^ ELD_50_/_mL_ NDV virulent virus at 84 days of age and survivability was assessed up to 14 dpc. All birds in the control group died by 4 dpc and one bird belonging to rR2B-FPCS-CIAV and rR2B-FPCS died by 7 dpc. All birds belonging to LaSota vaccine survived till the experimental study period.

Nine of the ten birds in the rR2B-FPCS-CIAV and rR2B-FPCS groups, and all of the ten birds of LaSota group survived the NDV challenge up to 14 dpc (Figure 7). In contrast, all the birds in the control challenge group displayed severe depression, whitish diarrhoea and paralysis with 100% mortality at 4 dpc.

#### 3.5.1. Virus Shedding Studies

**Figure 8:**
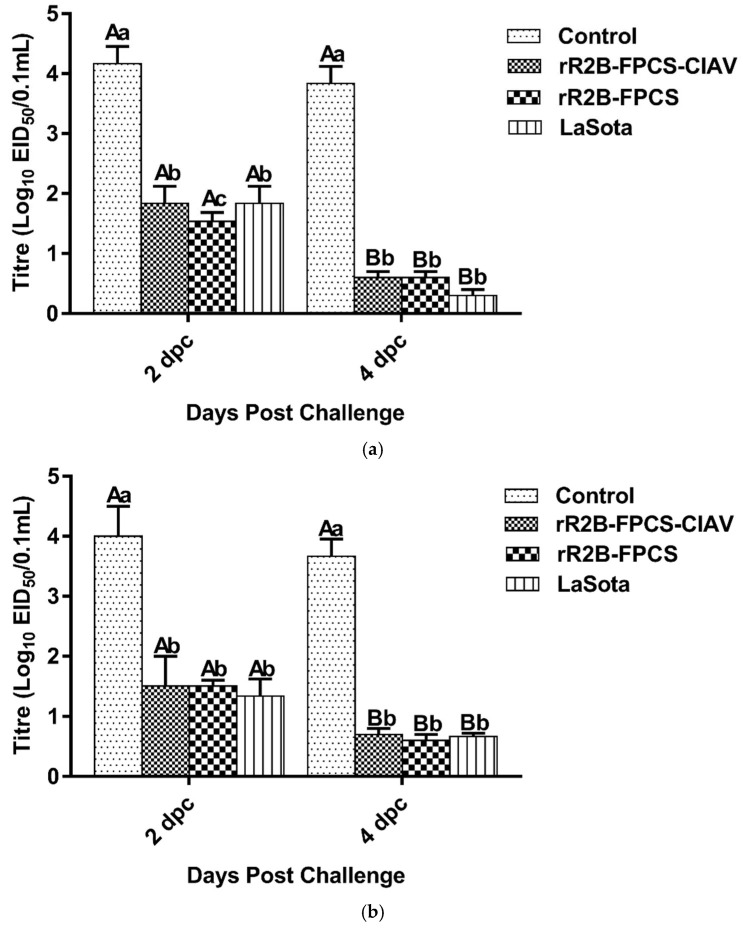
The effect of vaccination on virus shed in comparison to unvaccinated control group. Swabs (**a**) oropharyngeal and (**b**) cloacal were tested for isolation of virus on 2 and 4 dpc from the experimental birds. The virus shedding decreased significantly in the vaccinated groups by 4 dpc while the control birds were dead at the same time. Different capital letter superscripts indicate time effect (*p* < 0.0001) while the small letter superscripts indicate the treatment effect (*p* < 0.0001).

The oral and cloacal swabs were collected from control and vaccinated birds and were tested for the presence of NDV on 2 dpc and 4 dpc that were found to be positive. There was a significant reduction in the viral shedding at 4 dpc amongst all vaccinated birds as compared to control (in both oral and cloacal; *p* < 0.0001) group (Figure 8a,b).

#### 3.5.2. Haematology

The PCV, haemoglobin, TEC, TLC, PLC and PHC were estimated on 5, 10, 15, 20 and 25 dpc and the values are depicted in Table 1. The birds that were subjected to only CIAV infection showed a statistically significant decrease in all the haematological parameters compared to other groups analysed during different intervals. The haematological parameters of rR2B-FPCS-CIAV-vaccinated group did not reveal any statistically significant difference when compared to the uninfected control. 

#### 3.5.3. Gross and Histopathological Findings

**Figure 9:**
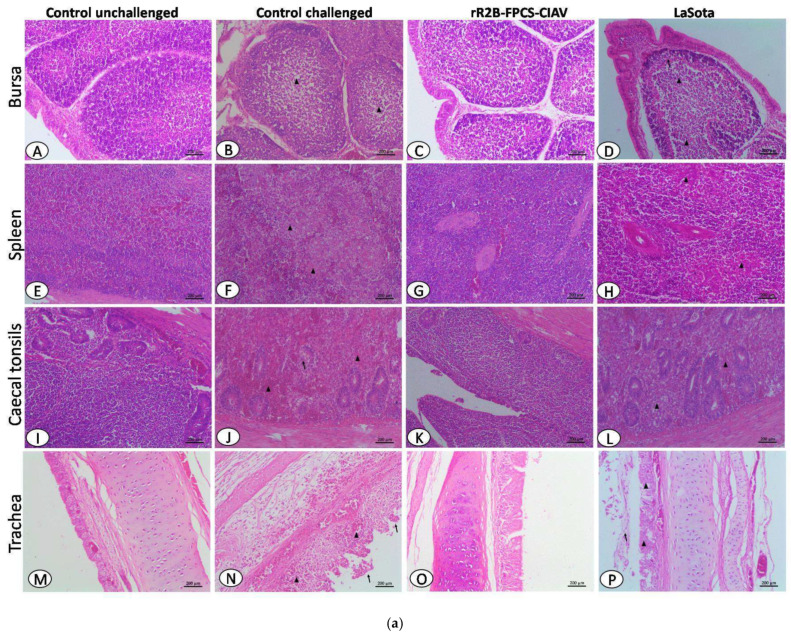

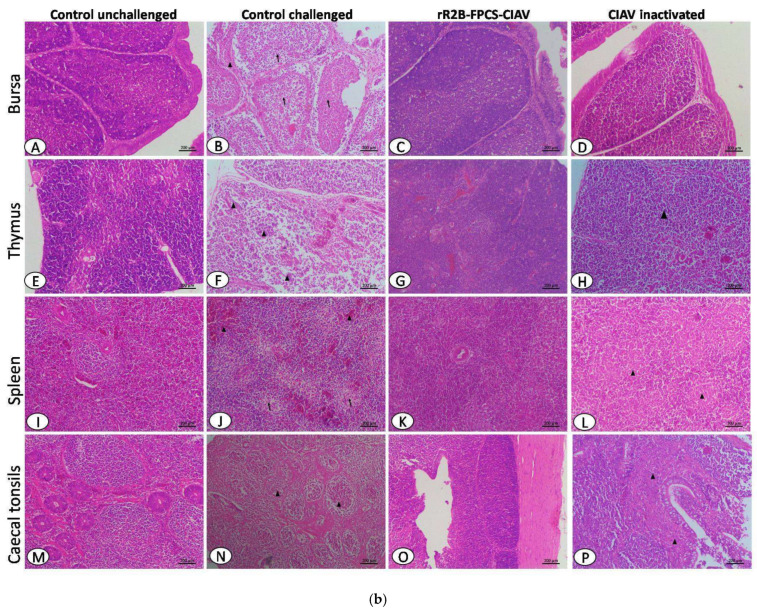
(**a**) Histopathological changes in various immune organs and trachea following virulent NDV challenge. A–D: bursa of Fabricius, H&E 10×, A: apparently normal bursa with intact follicles and plical epithelium, B: bursal follicles with severe lymphoid depletion in the medulla (arrow head), C: apparently normal bursa with intact follicles, D: thinning of cortex (arrow) and mild lymphoid depletion in the medulla (arrow). E–F: spleen, H&E 10×, E: apparently normal splenic architecture with intact PALS and PELS, F: severe lymphoid depletion with severe RE cell hyperplasia (arrow head), G: apparently normal splenic architecture, H: mild lymphoid depletion with RE cell hyperplasia (arrow head). I–L: caecal tonsils H&E 10×, I: apparently normal lymphoid aggregations in the caecal tonsils, J: severe lymphoid depletion with associated haemorrhages (arrow head) along with degeneration of the tonsillar cryptal lining epithelium (arrow), K: apparently normal lymphoid aggregations in the caecal tonsils, L: mild lymphoid depletion with RE cell hyperplasia (arrow head). M–P: trachea H&E 10×, M: apparently normal tracheal histoarchitecture, N: necrosis and denudation of tracheal lining epithelium (arrow) with severe inflammatory exudate and haemorrhages in the sub-mucosa (arrow head), O: hyperplastic lining epithelial cells, P: hyperplastic lining epithelial cells (arrow head) with denudation of mucosa into the lumen (arrow). (**b**) Histopathological changes in various immune organs following virulent CIAV challenge. A–D: bursa of Fabricius, H&E 10×, A: apparently normal bursa with intact follicles and plical epithelium, B: bursal follicles with severe lymphoid depletion in both cortex (arrow) and medulla with mild inter-follicular fibrosis (arrow head), C: apparently normal bursa with intact follicles and plical epithelium compared to control group, D: apparently normal bursal follicles with mild lymphoid depletion in the medulla. E–F: thymus, H&E 10×, E: apparently normal thymic corpuscles with intact cortex and medulla, F: thymic corpuscles showing severe lymphoid depletion in both cortex and medulla (arrow head), G: apparently normal thymic architecture, H: mild lymphoid depletion in the thymic cortex (arrow head). I–L: spleen H&E 10×, I: apparently normal splenic architecture with intact PALS and PELS, J: multifocal areas of haemorrhages in the PALS area (arrow head) with lymphoid depletion (arrow), K: spleen showing mild lymphoid depletion, L: moderate lymphoid depletion with RE cell hyperplasia (arrow head). M–P: caecal tonsils H&E 10×, M: apparently normal lymphoid aggregations in the caecal tonsils, N: moderate lymphoid depletion of the caecal tonsils (arrow head), O: apparently normal lymphoid cell aggregation in the caecal tonsils, P: very mild lymphoid depletion in the caecal tonsils (arrow head). Scale bar 200 µm.

In the virulent NDV challenge, the birds of NDV-infected group showed petechial haemorrhages in the proventricular glands, haemorrhages in the caecal tonsils, necrosis of the gut-associated lymphoid tissues in the intestines and mottling of spleen, and the trachea was highly congested with the mucosa containing necro-caseous exudate. All the other groups did not reveal any gross changes in visceral and immune organs. In the case of the virulent CIAV challenge, no abnormalities were detected in any of the examined lymphoid and visceral organs of the control group, rR2B-FPCS-CIAV-vaccinated birds and CIAV-inactivated vaccine group. In the CIAV-infected group, thymus and bursa revealed atrophic changes with the presence of pinpoint haemorrhages on the surface and the spleen appeared mottled. These findings correlated well with histopathological lesions (Figure 9a,b).

## 4. Discussion

Newcastle disease and chicken infectious anaemia infection in chickens evoke considerable interest among researchers and poultry industry stakeholders due to heavy economic losses. The unavailability of a suitable live attenuated vaccine for CIAV infection due to the refractory growth of the virus in embryonated chicken eggs and cell culture systems, combined with the suboptimal immunogenicity generated by the inactivated and subunit vaccines prompted us to develop a vaccine candidate against CIAV using NDV as a vector, which has a propensity to multiply in both embryonated chicken eggs and established cell lines to high titres. In this context, the backbone of the virus plays a major role in determining the immunogenicity and the protective efficacy of the vaccine. NDV strain R2B, used as a backbone in this study, has been utilised for several decades in endemic countries as a booster vaccine against NDV due to the moderate virulence accorded to young chicks that are less than 6 weeks old. Hence, in an earlier study, our research group changed the FPCS region of the virus to a less virulent motif so that the virus can be used as a primary vaccine in birds [8]. The recombinant virus generated with the altered backbone was able to confer immune response and protection against avian reovirus and rabies virus [9,10]. 

The immune response accorded against CIAV is encoded in the VP1 gene of the virus which is involved in capsid formation [29]. The proper conformation of VP1 protein is aided by the VP2 protein which shows phosphatase activity and a synchronous synthesis of both VP1 and VP2 proteins in the same cell [4,18]. In the present work, both the VP1 and VP2 proteins were expressed from a single transcription cassette between the P and M genes of NDV. The optimal expression of foreign proteins from NDV has been achieved earlier using this site by us and other groups [24]. In order to achieve expression of the full complement of both the foreign genes with equimolar levels, the FMDV-2A protease gene was introduced between the VP2 and VP1 genes in the transcription cassette. The FMDV-2A protease is a self-cleaving protease used by earlier groups in generating functional proteins when proteins are expressed in a fused form [30,31]. The equitable amounts of foreign proteins expressed is evident by the uniform levels of fluorescence found in Vero cells against NDV and CIAV proteins. The immunoblot indicates the presence of both the viral proteins although with varying intensities probably due to the differed quantum of antibodies generated against these proteins in the primary antibody that was used to probe the blot. Furthermore, the molecular and biological characterisation of the recombinant virus revealed that the virus has been sufficiently attenuated, as evidenced by a reduction in viral titres and the pathogenicity indices which indicate the lentogenic nature of the virus.

Live NDV-vectored poultry vaccines are efficient in stimulating both humoral and cellular immune responses [9,24]. In this study, the bivalent vaccine candidate developed for the first time against NDV and CIAV was compared with the NDV backbone containing altered FPCS amino acid motif to assess the level of immune responses generated by both these viruses. Immunisation of birds was carried out via intranasal, oral routes as this virus has lost its virulence due to the change in FPCS region, as reported earlier [8]. A detectable immune response was seen in birds after primary vaccination which significantly increased after the first and second boosters [24,32,33,34]. Moreover, antibodies against CIAV also showed a similar trend as described [13]. In the CIAV IDEXX ELISA, an S/N ratio of ≥0.6 is considered negative. In the present study, the ratio was <0.8 in the beginning, with the ratio becoming <0.5 from 35 days of age of bird and continuing until 49 days of age and hence a second booster was given at 56 days of age to maintain the titre of antibodies until challenge.

The lymphocyte transformation test is widely used to evaluate the CMI responses. In the present study, the lymphoproliferative responses of the vaccine groups were compared with the controls. Lymphocytes from the immunised groups proliferated in response to recombinant NP protein of NDV and recombinant VP2/VP1 antigen of CIAV expressed in yeast, indicating successful activation of antigen-specific immune responses as reported earlier [9,13].

Cytokines play a key role in prevention of viral infections [35]. IL-12 acts as an immunomodulator and adjuvant [36] (Peng Xie et al., 2020) and in the present study it was able to induce increased levels of CD4^+^ and CD8^+^ T cell subsets in rR2B-FPCS-CAV group, as evidenced by flow cytometry at 21, 35 and 63 days of age of the bird. IL-12 also induced IFN-γ production in this study and enhanced the CMI response as reported earlier [37]. Further, IFN-γ induced Th-1 response along with adjuvant activity as evidenced by Xin Yang et al., 2020 [38]. IL-4 expression induced increased antibody production in the vaccinated groups.

The protective efficacy studies reveal that the rR2B-FPCS-CAV-vaccinated group gave 90% protection similar to the rR2B-FPCS group against the virulent NDV challenge. In contrast, all the birds in the control unvaccinated group died with the NDV challenge. All the birds of the vaccinated and unvaccinated control groups survived the CIAV challenge, indicating the lower lethality of the virus as compared to NDV but with severe gross and histopathological lesions with altered haematological parameters.

The birds that were subjected to only CIAV infection showed significant decrease in all the haematological parameters compared to other groups analysed during different intervals; the decrease in the PCV, haemoglobin and TEC parameters is mainly attributed to the destruction of erythroid cells by CIAV [39]. Peripheral lymphocyte count in the present study was lower in CIAV infection compared to other groups; this indicates immunosuppression and the cause is attributed to the destruction of progenitor cells of T lymphocyte series in the thymus by CIAV [40]. In the vaccinated groups, the haematological parameters were apparently normal and this clearly indicates that the vaccine virus hinders the replication of the challenge virus in the target organs, preventing a clinical disease. 

In the present study, the chickens infected with CIAV revealed reduction in the size of the immune organs examined as compared to the non-infected control birds. The above findings are in agreement with the earlier reports wherein CIAV infection causes reduction in the progenitor cells of erythroid and myeloid series leading to anaemia and immunosuppression [39,41].

In the case of birds challenged with NDV, grossly there were haemorrhages in the proventricular glands and caecal tonsils, necrosis of the gut-associated lymphoid tissues in the intestines and mottling of spleen, and the trachea was highly congested with the mucosa containing necro-caseous exudate. The above changes were reported earlier in naturally infected cases of NDV [42]. All the other groups in the present study did not reveal any gross changes in visceral and immune organs, which hints that the vaccine ameliorates the effect of the challenge virus. The microscopic lesions are of severe lymphoid cell necrosis with reticuloendothelial (RE) cell hyperplasia in the spleen, bursa and thymus, which correlated with the lesion score; the findings are in agreement with the earlier studies [42,43]. The lesion scores in rR2B-FPCS-CIAV-vaccinated group, control group, and LaSota vaccine group were similar, which indicates that the challenge virus could not inflict pathology in the vaccinated birds owing to the protection offered by the vaccine. 

Amongst all progenitor cells, bone marrow hemocytoblasts, thymic T cell precursors and dividing T cells comprise the cell population susceptible to CIAV infection [44]. The damage to progenitor cells results in lymphoid cell depletion in the immune organs, especially thymus, caecal tonsils and Harderian glands. The above findings are in line with the histological findings of the present study wherein there was severe cellular depletion of lymphoid cells in various immune organs of the chickens, especially thymus with associated reticular cell hyperplasia.

In summary, a bivalent vaccine candidate against NDV and CIAV was generated for the first time by reverse genetics. The FMDV-2A protease used in this study was able to generate two functional immunogenic proteins of CIAV needed to elicit the requisite immune responses in birds against CIAV infection. Both humoral and cell-mediated immune responses against NDV and CIAV were generated to sufficient levels and accorded 90% protection in a challenge study. The gross and histopathological lesions along with haematological parameters in chickens indicated its corroboration with the immune responses. The current technique of vaccine production would help the researchers to circumvent the more cumbersome method of generating vaccines against CIAV due to the refractory growth of the virus in established cell lines and embryonated chicken eggs.

## Figures and Tables

**Table 1:** Effect of CIAV inoculation on packed cell volume (PCV), total erythrocytes count (TEC), total leukocyte count (TLC), peripheral lymphocyte count (PLC), peripheral heterophil count (PHC) and haemoglobin (Hb%) in CIAV-challenged chicks.

Parameters	Group	CIAV Post Inoculation Days				
		5	10	15	20	25
**PCV %**	**Control unchallenged**	29.66 ± 1.52 ^a^	31.33 ± 1.52 ^a^	30.20 ± 0.72 ^a^	30.23 ± 0.68 ^a^	29.83 ± 0.76 ^a^
	**Control challenged**	29.33 ± 0.57 ^a^	26.33 ± 1.52 ^b^	21.83 ± 1.75 ^b^	24.30 ± 0.60 ^b^	25.73 ± 0.64 ^b^
	**rR2B-FPCS-CIAV**	29.50 ± 0.50 ^a^	29.66 ± 2.08 ^a^	30.10 ± 0.85 ^a^	30.06 ± 0.90 ^a^	30.30 ± 0.60 ^a^
	**CIAV-Inactivated**	31.67 ± 0.57 ^a^	29.66 ± 1.52 ^a^	29.13 ± 0.80 ^a^	29.16 ± 1.25 ^a^	30.16 ± 0.76 ^a^
**TEC (Millions/mm^3^)**	**Control unchallenged**	3.2 0 ± 0.10 ^a^	3.76 ± 0.10 ^a^	3.46 ± 0.10 ^a^	3.21 ± 0.10 ^a^	3.11 ± 0.09 ^a^
	**Control challenged**	1.90 ± 0.05 ^b^	1.96 ± 0.01 ^b^	1.94 ± 0.14 ^b^	1.95 ± 0.02 ^b^	1.91 ± 0.07 ^b^
	**rR2B-FPCS-CIAV**	2.95 ± 0.03 ^b^	2.94 ± 0.04 ^c^	2.84 ± 0.10 ^c^	2.84 ± 0.06 ^c^	2.90 ± 0.09 ^c^
	**CIAV-Inactivated**	2.11 ± 0.07 ^b^	1.94 ± 0.04 ^b^	1.97 ± 0.12 ^b^	1.92 ± 0.07 ^b^	1.94 ± 0.05 ^b^
**TLC** **(×10^3^/mm ^3^)**	**Control unchallenged**	23,452 ± 97.51 ^a^	24,066 ± 57.30 ^a^	22,818 ± 76.37 ^a^	29,350 ± 50.00 ^a^	29,517 ± 76.37 ^a^
	**Control challenged**	11,399 ± 99.00 ^b^	10,283 ± 76.37 ^b^	9790 ± 85.44 ^b^	10,157 ± 98.14 ^b^	10,817 ± 76.37 ^b^
	**rR2B-FPCS-CIAV**	20,317 ± 76.36 ^c^	19,733 ± 76.37 ^c^	18,853 ± 95.04 ^c^	19,543 ± 92.91 ^c^	19,787 ± 80.82 ^c^
	**CIAV-Inactivated**	18,483 ± 28.87 ^d^	16,066 ± 57.73 ^d^	16,383 ± 76.37 ^d^	17,217 ± 76.37 ^d^	17,717 ± 57.73 ^d^
**PLC %**	**Control unchallenged**	45.67 ± 0.57 ^a^	46.83 ± 0.76 ^a^	45.23 ± 0.68 ^a^	46.16 ± 1.25 ^a^	47.20 ± 1.31 ^a^
	**Control challenged**	21.67 ± 1.52 ^b^	22.16 ± 1.25 ^b^	21.16 ± 1.25 ^b^	23.20 ± 0.72 ^b^	26.23 ± 1.36 ^b^
	**rR2B-FPCS-CIAV**	42.33 ± 1.15 ^c^	39.16 ± 0.76 ^c^	40.06 ± 0.90 ^c^	40.30 ± 0.60 ^c^	42.30 ± 1.47 ^c^
	**CIAV-Inactivated**	43.67 ± 0.57 ^c^	39.73 ± 0.64 ^c^	39.10 ± 0.85 ^c^	39.10 ± 1.15 ^c^	40.20 ± 0.72 ^c^
**PHC %**	**Control unchallenged**	53.67 ± 1.52 ^a^	53.10 ± 0.85 ^a^	54.16 ± 0.76 ^a^	54.20 ± 0.72 ^a^	53.20 ± 0.72 ^a^
	**Control challenged**	74.33 ± 2.0 ^b^	75.13 ± 1.20 ^b^	76.20 ± 1.31 ^b^	76.16 ± 0.76 ^b^	73.10 ± 1.15 ^b^
	**rR2B-FPCS-CIAV**	57.67 ± 1.52 ^a^	61.53 ± 1.85 ^c^	59.26 ± 0.64 ^c^	59.20 ± 1.31 ^c^	57.00 ± 0.90 ^c^
	**CIAV-Inactivated**	54.0± 2.60 ^a^	58.16 ± 1.25 ^c^	60.20 ± 0.72 ^c^	60.00 ± 0.80 ^c^	59.03 ± 0.95 ^c^
**Hb (g %)**	**Control unchallenged**	9.77 ± 0.20 ^a^	11.33 ± 1.52 ^a^	9.76 ± 0.15 ^a^	9.60 ± 0.10 ^a^	9.70 ± 0.10 ^a^
	**Control challenged**	9.80 ± 0.10 ^b^	8.23 ± 0.15 ^b^	7.60 ± 0.10 ^b^	8.20 ± 0.10 ^b^	8.50 ± 0.30 ^b^
	**rR2B-FPCS-CIAV**	10.30 ± 0.20 ^b^	10.16 ± 0.76 ^c^	11.20 ± 0.20 ^c^	10.23 ± 0.25 ^c^	10.36 ± 0.32 ^c^
	**CIAV-Inactivated**	10.20 ± 0.20 ^b^	9.80 ± 0.10 ^c^	9.70 ± 0.15 ^d^	9.60 ± 0.10 ^c^	9.50 ± 0.20 ^c^

The values (Mean ± SD) having at least one common superscript (small letters in columns for different groups on a particular day) does not differ significantly (*p* < 0.001) for a given parameter.

## Data Availability

All datasets generated for this study are included in the article.
